# Expression of *Otx* Genes in Müller Cells Using an *In Vitro* Experimental Model of Retinal Hypoxia

**DOI:** 10.1155/2021/6265553

**Published:** 2021-12-31

**Authors:** Claudio Azzolini, Simone Donati, Giovanni Micheloni, Vittoria Moretti, Roberto Valli, Francesco Acquati, Lucy Costantino, Fulvio Ferrara, Davide Borroni, Elias Premi, Francesco Testa, Francesca Simonelli, Giovanni Porta

**Affiliations:** ^1^Department of Medicine and Surgery, University of Insubria, Varese-Como, Italy; ^2^Ophthalmology Unit, ASST Sette-Laghi, Varese, Italy; ^3^Genomic Medicine Research Center, Department of Medicine and Surgery, University of Insubria, Varese-Como, Italy; ^4^Department of Biotechnology and Life Science, University of Insubria, Varese-Como, Italy; ^5^Department of Molecular Genetics, Centro Diagnostico Italiano, Milano, Italy; ^6^Fondazione Banca Degli Occhi Del Veneto Onlus, Zelarino, Venezia, Italy; ^7^Department of Doctoral Studies, Riga Stradins University, Riga, Latvia; ^8^Eye Clinic, Multidisciplinary Department of Medical Surgical and Dental Sciences, University of Campania Luigi Vanvitelli, Naples, Italy

## Abstract

**Introduction:**

Müller glial cells typically activate to react to hypoxic tissue damage in several retinal diseases. We evaluated the *in vitro* response to a hypoxia-mimicking stimulus on the expression of a set of genes, known to contribute to eye morphogenesis and cell differentiation.

**Materials and Methods:**

A MIO-M1 Müller cell line was cultured in a hypoxia-mimicking environment by the addition of cobalt chloride to the culture medium, followed by a recovery time in which we mimic restoration from the hypoxic insult. The HIF-1*α* protein and VEGF-A gene expression were quantified to verify the induction of a hypoxia-like state.

**Results:**

Among the genes under study, we did not observe any difference in the expression levels of *Otx1* and *Otx2* during treatment; conversely, *Otx1* was overexpressed during recovery steps. The VEGF-A gene was strongly upregulated at both the CoCl_2_ and recovery time points. The transactivated isoform (TA) of the *TP73* gene showed an overexpression in long-term exposure to the hypoxic stimulus with a further increase after recovery. *Discussion*. Our molecular analysis is able to describe the activation of a set of genes, never before described, that can drive the response to a hypoxia-like status. The improved comprehension of these cellular events will be useful for designing new therapeutical approaches for retinal pathologies.

## 1. Introduction

Müller cells represent the most important glial population in the retina, with both structural and functional roles in retinal homeostasis [1]. Under physiological conditions, Müller cells provide an orientated scaffold and metabolic support to neuronal activity; they maintain the integrity of the blood-retinal barrier [2, 3] and regulate the fluid homeostasis and neovascularization (NV) processes. Several pathological alterations or injuries can activate these cells, causing morphological and/or molecular alterations [4].

In several retinal diseases, such as diabetic retinopathy, retinal vascular occlusion, or retinal detachment, the reduction of vascular perfusion is responsible for the hypoxia and/or ischemia of intraocular tissues [5]. This results in the activation of cellular mechanisms such as neovascular proliferation, intraretinal cell migration, and extravascular leakage of fluids secondary to cytokine release, affecting different retinal cell types [6]. These events, defined in terms of gliosis, may contribute to intraretinal edema formation and retinal tissue degeneration [7].

Müller cells produce a vascular endothelial growth factor (VEGF) in response to hypoxic stresses to improve vascularization and vascular permeability [8]. In this setting, it plays multiple significant roles: in the early phases of oxidative or hypoxic injury, VEGF protects neuronal cells from apoptosis and leads to ischemic preconditioning, i.e., an adaptive protective response to ischemia caused by brief ischemic events. In later stages, it instead alters the expression of several genes affecting the peroxynitration balance by inducing the expression of the inducible nitric oxide synthase (*iNOS*), which leads to retinal inflammation, neovascularization, vascular leakage, and key pathological changes in hypoxic tissues [9]. In addition to its role on the vasculature, VEGF is an essential neurotrophic factor for the survival of photoreceptors and Müller cells, in an autocrine process that controls the proapoptotic *Bax* gene and its transcriptional activator *p53* [10].

The *Otx1* and *Otx2* genes code for two transcription factors, OTX1 and OTX2, respectively [11], which are involved in cell identity specification and differentiation and positioning of the bodily axis during the embryological development of the neuroectoderm, from which originates the adult central nervous system [12, 13]. *Otx* genes are essential in developing the eye, due to their expression in the optic vesicle and retinal progenitor cells (RPCs) [14], and *Otx2* is especially expressed in postmitotic neuroblast, which differentiates into all retinal cell types. In the adult retina, *Otx2* expression has been observed in bipolar interneurons and photoreceptors [15–17], where it controls the expression of several opsin genes [18, 19].

*Otx1* and *Otx2* mutant mice showed no retinal pigmented epithelium development [20], while *Otx2* mutations are associated with several eye defects such as microphthalmia or the lack of a lens, cornea, and iris [14].

In a previous study on samples of hypoxic retinal tissue from patients affected by proliferative vitreoretinopathy (PVR) secondary to retinal detachment, we observed an abnormal expression of both *Otx1* and *Otx2* associated with an increased severity of the disease in *Otx2* expressing samples [21]. More recently, these two genes have also been involved in physiological and pathological functions in adult tissues, such as sinonasal mucosae [22–25], the pituitary [26–28], and the mammary gland [29–31].

The *p53, TP63*, and *TP73* genes belong to the *p53* family and code for two isoforms, one containing the transactivation domain (TA) and the other truncated (Δ*N*). Either *p53* or the TA isoforms of the other two genes regulate the stability and activity of HIF and are known to be involved in defensive mechanisms, especially against oxidative stresses, a typical feature of hypoxia [32–34].

The *TP73* gene product is also essential for proper retinal vasculature formation [35], and, contrasting with *TP53* and *TP63*, there are no reports on the *TP73* and *Otx* genes.

The aim of our study is to evaluate the activation and modulation of the abovementioned set of genes involved in hypoxia response and differentiation in Müller cells treated with CoCl_2_*in vitro*.

## 2. Materials and Methods

### 2.1. Cell Line

The MIO-M1 cell line derives from the spontaneous immortalization (at least 100 divisions) of Müller cells obtained from an eye of a 68-year-old female corneal donor, 36 hours after death [36]. They grow in adhesion in Dulbecco's Modified Eagle Medium (DMEM) supplemented with 10% fetal bovine serum (FBS), 1% L-glutamine, and 1% penicillin/streptomycin (complete medium).

### 2.2. Analysis of the Morphology of MIO-M1 Cells

The morphology of the cells was evaluated with bright-field microscopy using an OLYMPUS IX51 inverted microscope (OLYMPUS, Shinjuku, Tokyo, Japan). The physiological morphology was compared with that described by Limb's research group [37].

### 2.3. Cell Culture Preparation for CoCl_2_ Treatment

MIO-M1 cells were cultured as described in the previous paragraph. Upon confluence, the cells were washed twice with sterile PBS (Phosphate Buffered Saline) and detached from the flask using trypsin-EDTA diluted in sterile PBS (1 : 1 ratio).

After a cell count in Trypan Blue through a Luna Cell Counter, to evaluate their viability, the Müller cells were seeded in new flasks.

The treatment was conducted on the cells at four passages after thawing, and generally, the cells were cultured for no more than five to six passages.

### 2.4. Cobalt Chloride Treatment

CoCl_2_ at a final concentration of 100 *µ*M, dissolved in culture-grade water, was used to mimic the hypoxic conditions. The treatment was performed in six-well plates seeded with 25000 cells/well. Before the addition of CoCl_2_, cells were maintained for 24 h in DMEM without FBS, to synchronize the cell cycle. Each experiment was performed in triplicate.

**Figure 1:**
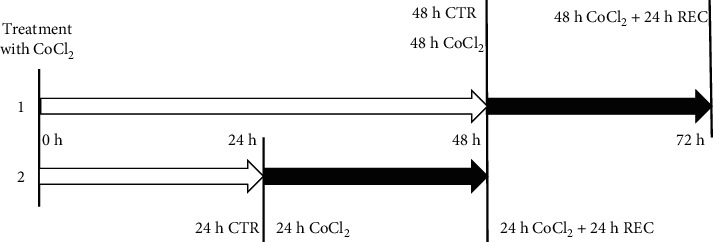
Schematic representation of two CoCl_2_ treatments. (1) Exposure of MIO-M1 cells to 100 *µ*M CoCl_2_ for 48 h followed by 24 h of recovery in a complete medium (DMEM + 10% FBS + 1% L-Glut + 1% Pen/Strep). (2) 24 h exposure to 100 *µ*M CoCl_2_ followed by 24 h of recovery. CTR: control sample. White arrow: medium added with CoCl_2_. Black arrow: medium without CoCl_2_. Black vertical lines indicate timepoints of cell collection.

The cells were exposed to CoCl_2_ for 24 and 48 hours (samples 24 h CoCl_2_ and 48 h CoCl_2_, respectively); after each exposure, a recovery time of 24 hours followed (REC), in which the cells were cultured in the complete medium without CoCl_2_ (samples 24 h CoCl_2_ + 24 h REC and 48 h CoCl_2_ + 24 h REC, respectively) (Figure 1).

In each phase, an aliquot of cells supplemented only with culture-grade water was collected in 50 mL tubes, to evaluate the effect of the solvent on the cell culture.

**Figure 2:**
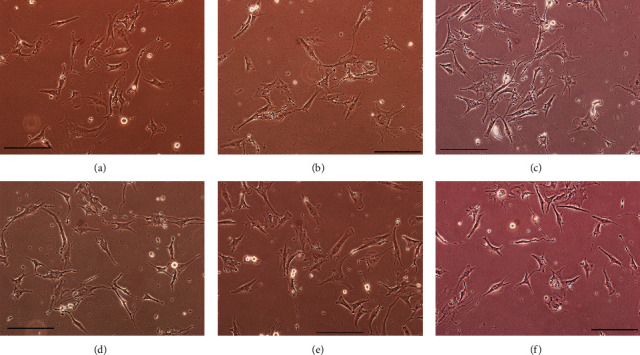
Morphological analysis of MIO-M1 Müller cells in different experimental conditions. (a) CTR 24 h; (b) 24 h CoCl2; (c) 24 h CoCl_2_ + 24 h REC; (d) CTR 48 h; (e) 48 h CoCl2; and (f) 48 h CoCl2 + 24 h REC. Black bar: 100 *μ*M.

Bright-field images were acquired for each sample at each time point (Figure 2).

The cells were centrifuged at 1200 rpm for five minutes, the supernatant was then discarded, and the resulting cellular precipitate (pellet) was resuspended in 1 mL of complete medium. The cells were then counted as previously described and split into two aliquots (500 *µ*L each) to perform further analysis.

### 2.5. Protein Extraction and Quantification

An initial aliquot of treated cells was used to extract proteins and to evaluate the levels of HIF-1*α* (hypoxia-inducible factor 1 subunit alpha) protein, whose amount is typically increased in response to hypoxic conditions.

The cells were centrifuged at 1200 rpm for five minutes, and the obtained pellet was lysed using NP40 (Thermo Fisher Scientific, Waltham, Massachusetts, USA) supplemented with 1 mM PMSF (phenylmethylsulphonyl fluoride) and a protease cocktail inhibitor (Sigma-Aldrich, St. Louis, Missouri, USA) for 30 min on ice and vortexed every 10 minutes. The lysed material was centrifuged at 13000 rpm for 10 minutes at 4°C, and the supernatant was collected for further analysis.

The proteins extracted were quantified using the Bradford reagent (Sigma-Aldrich, St. Louis, Missouri, USA).

### 2.6. SDS-PAGE

Next, 200 *μ*g of proteins were diluted with the Laemmli buffer, boiled for 10 min, and loaded on SDS-8% polyacrylamide gel (Sodium Dodecyl Sulphate, Sigma-Aldrich, St. Louis, Missouri, USA). SDS-polyacrylamide gel electrophoresis was performed with a running buffer (Tris-HCl 25 mM, glycine 0.2 M, SDS 0.1%) at a 110 V constant voltage. Proteins were wet-transferred from the gel to a polyvinylidene fluoride membrane (PVDF Immobilon-P, Millipore) in a cold chamber (+4°C), for 1 h 30 min with a blotting buffer (glycine 0.15 M, Tris-HCl 25 mM, and methanol 10% in ultrapure water) and at a 250 mA constant current. The membrane was stained with Ponceau S solution (Sigma-Aldrich, St. Louis, Missouri, USA) to control the effective transfer of proteins, then washed five times with PBS-T (NaCl 130 mM, KCl 2.6 mM, Na_2_HPO_4_ 8.4 mM, KH_2_PO_4_ 1.4 mM, pH 7.4, and Tween 20 at 0.1%) and incubated with a blocking solution (5% milk in PBS-T) for 2 h, to mask aspecific sites of the membrane.

### 2.7. Western Blot (WB) Analysis of HIF-1*α* Protein Levels

The PVDF membranes were incubated with HIF-1*α* and *β*-actin primary antibodies, followed by horseradish peroxidase-conjugated secondary antisera (Table 1). The primary antibodies were diluted in a blocking solution and incubated overnight at +4°C. The membrane was then washed five times with PBS-T; secondary antibodies diluted in blocking solution were added for 1 h at room temperature and, and after five washes with PBS-T, they were ready to be analyzed.

The Clarity Max™ Western ECL Substrate kit (Bio-Rad, Hercules, California, USA) was used to visualize the chemiluminescence of our proteins of interest. The membrane was exposed to a photographic film that was further processed with commercially available developer and fixer solutions (Kodak). Images were obtained by scanning the developed film, and the abundance of proteins of each band was evaluated by densitometric analysis with the ImageJ-NIH image software. The effect of CoCl_2_ treatment on HIF-1*α* protein levels was expressed as the fold change variation of the optical density (OD), expressed in arbitrary units of HIF-1*α* signals, normalized to the respective loading control *β*-actin.

### 2.8. RNA Isolation and Reverse Transcription

The total RNA was extracted from a second aliquot with the EuroGold Total RNA Mini Kit (Euroclone, Milan, Italy) and subsequently quantified using a NanoDrop ND 1000 spectrophotometer (Thermo Fisher Scientific, Waltham, Massachusetts, USA), following the manufacturer's instructions.

Subsequently, 2 *μ*g of RNA was reverse-transcribed using the High-Capacity cDNA Kit (Applied Biosystems, Foster City, California, USA) following the protocol provided by the developer. Real-time PCR (qRT-Polymerase Chain Reaction) was performed by SYBR®-Green technology using the ABI Prism 7000 apparatus (Applied Biosystems, Foster City, California, USA).

### 2.9. Gene Expression by qRT-PCR

Gene expression analyses were performed on 20 ng cDNA in a 25 *μ*L reaction, following this recipe: 12.5 *μ*L 2xMasterMix, 1 *μ*L of each primer, 20 ng cDNA, and nuclease-free water up to 25 *μ*L.

The thermocycler program consisted of an initial hot start cycle at 50°C for two minutes and 90°C for 10 minutes, followed by 40 cycles at 95°C for 15 seconds and 60°C for one minute, and a final cycle at 60°C for one minute. Each reaction was performed in triplicate. Negative controls (PCR mix without the addition of cDNA) were analyzed for each reaction. Human *β*-actin was used as an endogenous control to normalize gene expression levels.

### 2.10. Molecular Characterization and Gene Expression

We firstly evaluated the expression levels of different genes in untreated MIO-M1, to characterize the expression profile of these cells. We analyzed the expression of genes of the homeobox (*Otx1*, *Otx2*, and *Otx3*) and *p53* families (*TP53*, *TP63*, and *TP73*), VEGF-A and *WNT1*.  Table 2 summarizes the primers used. Each primer pair was tested to obtain the optimal concentration and annealing temperature. The geometrical efficiency of the reactions was assessed by a visual comparison of the amplification curves of our samples.

Threshold cycles were used to calculate the 2^−ΔCt^ values that give us the fold change of the expression of these genes compared to the reference actin in untreated cells.

Conversely, the ΔΔCt method was used to evaluate the effect of the treatment on the expression levels of these genes. This method allowed for obtaining the 2^−ΔΔCt^, a value that describes the fold change of the gene expression in treated vs. control samples, normalized to *β*-actin.

### 2.11. Statistical Analysis

Data are expressed as mean ± SD for both western blot and qRT-PCR analysis. Statistical analysis has been conducted through a one-way ANOVA test and Tukey's multiple-comparison test. Results are considered significant with *p* < 0.05.

## 3. Results

### 3.1. MIO-M1 Morphology

MIO-M1 cells in culture tend to spread over the entire surface of the plate, showing a bipolar morphology, cytoplasmic projections, and a rough membrane appearance. As we can see from the images in Figure 2, CoCl_2_ treatment does not macroscopically affect cell morphology after either 24 h (Figure 2(b)) or 48 h (Figure 2(e)).

### 3.2. HIF-1*α* Protein Level Increases after CoCl_2_ Exposure

**Figure 3:**
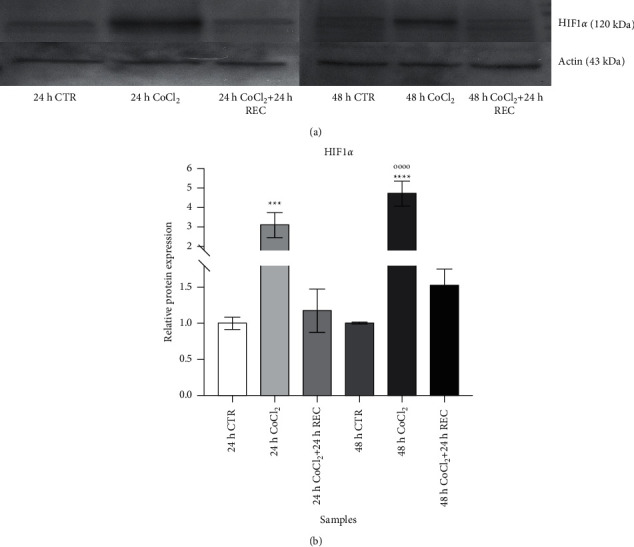
Analysis of HIF-1*α* and *β*-actin protein expression levels by western blot evaluated in MIO-M1 cells at different time points of treatment. (a) Representative immunoreactive bands for HIF-1*α* (top; 120 kDa) and *β*-actin (bottom; 43 kDa). For each sample, 200 *μ*g of protein was loaded on SDS-8% polyacrylamide gel. (b) Relative expression of HIF-1*α* protein, normalized to the respective *β*-actin, is represented as mean ± SD of three repeated experiments. ^*∗∗∗*^*p* < 0.001 (24 h CoCl_2_ vs. 24 h CTR); ^*∗∗∗∗*^*p* < 0.0001 (48 h CoCl_2_ vs. 24 h CTR); and *p* < 0.0001 (48 h CoCl_2_ vs. 48 h CTR) by one-way ANOVA and Tukey's post hoc test.

To confirm the induction of hypoxic stimulus in our experimental setting, we evaluated the HIF-1*α* protein amount by WB at each time point of treatment (Figure 3). The HIF-1*α*-specific primary antibody (Ab) detected a 120 kDa band, while *β*-actin Ab identified a band with a molecular weight of 43 kDa (Figure 3(a)).

As expected, the amount of HIF-1*α* protein significantly increased in response to CoCl_2_ treatment (24 h CoCl_2_ vs. 24 h CTR: *p* < 0.001; 48 h CoCl_2_ vs. 24 h CTR: *p* < 0.0001; and 48 h CoCl_2_ vs. 48 h CTR: *p* < 0.0001, evaluated by one-way ANOVA and Tukey's post hoc test), and the 24 h recovery phase after exposure to CoCl_2_ restored protein levels comparable to control samples (Figure 3(b)). Data in the graph (Figure 3(b)) represent the relative HIF-1*α* protein expression, normalized to the reference *β*-actin protein.

### 3.3. MIO-M1 Gene Expression Profile

We firstly evaluated the mRNA levels of a set of genes to characterize their expression profile in the untreated MIO-M1 cell lines.

**Figure 4:**
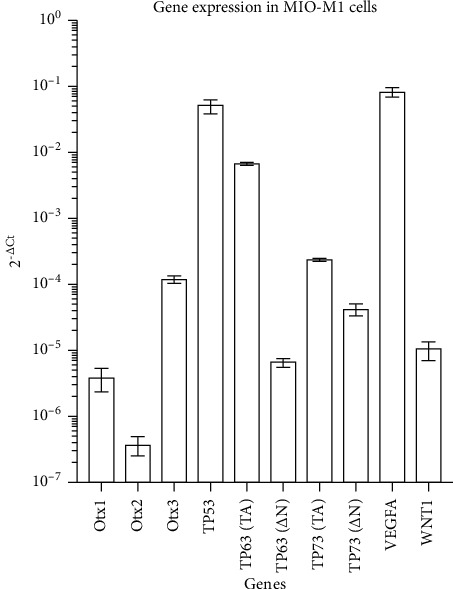
Molecular characterization of gene expression in untreated MIO-M1 cells. Each gene was normalized to *β*-actin expression level, to obtain a value representing the fold change between the two analyzed genes. On the *Y*-axis, the fold change value is reported. Each value is indicated as median ± SD.

Each gene expression level was normalized to *β*-actin, taken as a reference, applying the 2^−ΔCt^ method to obtain a fold change value representing how much each gene of interest is expressed in untreated MIO-M1 cells (Figure 4).

Genes belonging to the *Otx* family, such as *Otx1*, *Otx2*, and *Otx3*, were nearly completely turned off. VEGF-A is the highest expressed gene, as expected from data observed in the literature considering the pivotal role of Müller cells in VEGF-A regulation in the retina [36, 38].

Finally, genes belonging to the *p53* family showed a higher expression in the full-length TA isoforms, generally involved in tissue maintenance.

### 3.4. Expression of Otx1, Otx2, TP73, and VEGF-A after CoCl_2_ Treatment

**Figure 5:**
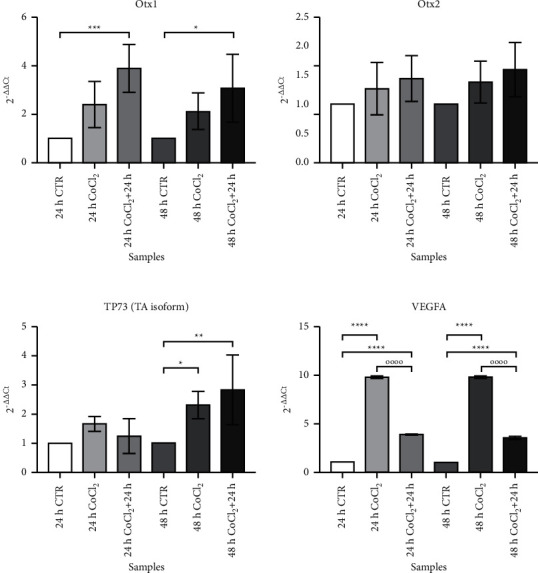
qRT-PCR on CTR- and CoCl_2_-treated MIO-M1 Müller cells. Each graph represents the expression levels of a single studied gene in different experimental conditions. All values are indicated as median ± SD. On the *Y*-axis, the fold change value is reported. Statistical analysis was performed by one-way ANOVA and Tukey's post hoc test. ^*∗*^*p* < 0.05; ^*∗∗*^*p* < 0.01; ^*∗∗∗*^*p* < 0.001; and ^*∗∗∗∗*^*p* < 0.0001.

We finally analyzed the gene expression levels of some of these genes by qRT-PCR after treatment with CoCl_2_. The data obtained are summarized in Figure 5, presenting fold change values for each gene, obtained through the 2^−ΔΔCt^ method, which allows for determining the relative expression of a gene of interest, normalized toward *β*-actin and in treated vs. untreated samples.

We observed a strong upregulation, i.e., higher mRNA production, of VEGF-A in all analyzed samples, with higher fold changes in response to CoCl_2_ (24 h CoCl_2_: 9.748 ± 0.143 and 48 h CoCl_2_: 9.782 ± 0.191, *p* < 0.0001) compared to the recovery phase (3.878 ± 0.077 and 3.521 ± 0.189 for 24 h and 48 h + 24 h, with a *p* < 0.0001).

*Otx2* expression levels did not show statistically significant modifications in response to the treatment or after the recovery time.

Interestingly, we observed an increased expression of the *Otx1* gene in cells undergoing a recovery time after exposure to CoCl_2_ for either 24 h or 48 h (24 h CoCl_2_ + 24 h REC: 3.878 ± 0.9842, *p* < 0.001; 48 h CoCl_2_ + 24 h REC: 3.076 ± 1,397, *p* < 0.05).

The TA isoform of the *TP73* gene was upregulated only in samples treated for 48 h, with a higher expression during the recovery phase compared with the treatment with only CoCl_2_ (48 h CoCl_2_: 2.301 ± 0.4676, *p* < 0.05; 48 h CoCl_2_ + 24 h REC: 2.825 ± 1.196, *p* < 0.01).

## 4. Discussion

Our research aimed to evaluate the expression modulation of genes involved in the response to stress and in differentiation, in Müller cells undergoing hypoxic stimulus, a typical condition of several retinal diseases. Cobalt chloride treatment can mimic hypoxia in two ways: cobalt inhibits the hydroxylation of a proline residue in the ODD (Oxygen-Dependent Degradation) domain of HIF-1*α* protein, thus preventing its recognition by the von Hippel–Lindau protein (pVHL) and the subsequent ubiquitination and degradation of HIF-1*α*, but it is also able to prevent the interaction between hydroxylated HIF-1*α* and pVHL [39, 40]. CoCl_2_ is also part of the vitamin B12 complex, and its accumulation causes ferroptosis-like cell death in neuronal cells. Moreover, it influences mitosis and the processes involved in preserving neuronal integrity [41].

Increased HIF-1*α* protein levels in response to CoCl_2_, but not in the recovery phase, confirmed the effectiveness of the treatment. The overexpression of VEGF-A in treated samples supported the activation of HIF-1*α* downstream pathways and the VEGF-A role in neuroprotection and as a survival factor [9–36].

The *TP73* gene product role in hypoxia and angiogenesis is more controversial. Its activity in enhancing defense against oxidative stresses, a typical feature of hypoxia, is well documented [31–34], and it is essential for proper retinal vasculature formation [35]. Sabapathy hypothesizes a bifunctional role of the TAp73 protein: in the initial stages of hypoxia, HIF-1*α* activates *TP73* expression and stabilizes the TAp73 protein that exerts a proangiogenic function activating downstream genes [42]. Once the vasculature is formed, TAp73 acts in a negative regulatory loop to turn off HIF-1*α* [42–44].

The observed upregulation of *TP73* after 48 h of treatment with CoCl_2_ may be associated with a response to prolonged hypoxic stimulus mediated by HIF-1*α* accumulation.

The further increase in expression observed in the recovery phase could be due to the HIF-1*α*-independent protective role exerted to overcome the hypoxic insult.

To our knowledge, this is the first report that evaluates *Otx1* and *Otx2* expression modulation in response to hypoxia in the MIO-M1 cell line.

Both genes are involved in eye and ear tissue development and maintenance in adults [14–36, 38–46]; thus, we wanted to investigate their behavior in Müller cells during hypoxic stimuli.

We have already reported the expression and modulation of these two genes in retinal samples of patients affected by PVR, observing better surgical and clinical outcomes in patients with higher *Otx1* expression compared with those with *Otx2* [17].

Previous studies on two mouse models of intestinal ischemia/reperfusion and DNBS-induced (DiNitroBenzene Sulfonic acid) colitis in the enteric nervous system (ENS) showed OTX2 protein mainly expressed in neurons of the ENS in association with the neuronal isoform of nitric oxide synthase (nNOS), while OTX1 presented a glial distribution and an association with the inducible NOS [47, 48].

In accordance with these previous experiments, in our experimental models, we did not observe a variation in *Otx2* expression levels during the hypoxic phase or in the recovery period, while *Otx1* was upregulated during both recovery phases.

Considering its role in cellular differentiation and development, we can hypothesize that the overexpression of *Otx1* in Müller cells under stress conditions is involved in cell gliosis, playing a role in the induction of proliferation and morphological processes observed in retinal pathological conditions [7]. One of the most relevant factors released from Müller cells under hypoxic conditions is the vascular endothelial growth factor (VEGF-A). VEGF-A supports the survival of endothelial cells and retinal neurons and may restrict glucose- and oxidative stress-induced damage in retinal vessels [49]. The effects of VEGF-A include vasodilation, revascularization, inflammation, and glial cell proliferation, which turn into a pathological pathway when VEGF-A levels exceed a threshold level. Retinal tissue degenerates due to tissue folds, edema, and atrophy, with a decrease in visual acuity and finally a loss of function of the eye. On the other side, according to ischemic stimuli, the *Otx1* and other gene pathways may control the neuroregenerative and neuroprotective VEGF-A function. Understanding how to modulate these events may allow us to direct Müller cell gliosis toward reparation of the injury, with visual function recovery, and surgical or medical therapy will show a better prognosis.

It is well known that teleost and chicken retinae maintain regenerative properties, mainly associated with the Müller cell ability to dedifferentiate and redifferentiate into any cell type of retinal tissue [50]. This ability is lost in mammals, even if Müller cells cultured *in vitro* exhibit neural stem cell characteristics [51] and can differentiate if properly stimulated [52].

However, most of the retinal degenerative conditions in humans are associated with an altered proliferation of Müller cells that lead to the formation of glial scars, as in proliferative vitreoretinopathy. Reactive gliosis has been described in different retinal pathologies, including age-related macular degeneration (AMD), diabetes, glaucoma, and retinal detachment.

The identification of genes conferring differentiation capacity, such as *Otx1*, and how they behave during the hypoxic response can lead us to a better understanding of the dual mechanism, neuroprotective and/or cytotoxic, of microglial Müller cells.

This knowledge can be helpful in modulating and treating retinal pathologies pointing to Müller cells as key targets for new drug therapies.

The main limitation of this study is the fact that we considered only Müller cells and no other subtypes of retinal cells or pigmented epithelium cells. All retinal layers are involved in maintaining the correct retinal physiology. However, our preliminary study *in vitro* on Müller cell genes in hypoxic conditions could represent a good first step for further studies.

## 5. Conclusions

In conclusion, our experimental model of mimicked hypoxia showed an interesting mechanism in Müller cells: *Otx1*, *Otx2*, and *TP73* gene expressions were altered after CoCl_2_ treatment of MIO-M1. The significant increase in *Otx1* levels in the recovery phase pointed to its involvement in processes activated after a hypoxic stimulus: we need to verify in which pathway *Otx1* plays a role and its association with vasoactive dynamics, as well as in inflammation and on its pathological or protective role in tissues. *TP73* upregulation may suggest an activation of mechanisms of defense usually caused by the accumulation of oxidative stresses in hypoxia.

## Figures and Tables

**Table 1:** Antibodies used to detect HIF-1*α* and *β*-actin protein levels and primary and secondary antibodies used in WB.

Antibody	Dilution	Host species	Source
HIF-1*α*	1 : 300	Mouse	Cell Signaling Technology (D5F3M)
*β*-Actin	1 : 1000	Goat	Abcam (ab8229)
Anti-mouse IgG-HRP	1 : 2000	Goat	Santa Cruz (sc2005)
Anti-goat IgG-HRP	1 : 5000	Donkey	Santa Cruz (sc2020)

**Table 2:** List of primers used in qRT-PCR experiments. Each primer sequence is written from the 5′ end to the 3′ end.

Gene	Fw (5′–3′)	Rv (5′–3′)
*ACTB*	CGCGAGAAGATGACCCAGAT	ACAGCCTGGATAGCAACGTACA
*Otx1*	TGCCGACTGCTTGGATTACA	GCCATGAGGCTTGGTCCTTA
*Otx2*	CGCAGCTAGATGTGCTGGAA	TCGACTCGGGCAAGTTGATT
*Otx3*	CCCCAAAGCTGAGAAGAGC	GCCACAGGAGTGATGGTCA
*TP53*	TCTTCTGTCCCTTCCCAGAA	AGAATGCAAGAAGCCCAGAC
*TP63 (TA isoform)*	TTTGAAACTTCACGGTGTGC	TGAGCTGGGGTTTCTACGA
*TP63 (*Δ*N isoform)*	GGTTGGCAAAATCCTGGAG	GGTTCGTGTACTGTGGCTCA
*TP73 (TA isoform)*	AACCAGACAGCACCTACTTCG	CGCCCACCACCTCATTATT
*TP73 (*Δ*N isoform)*	AAGCGAAAATGCCAACAAAC	AGGCTCCGCAGCTAGTGA
*VEGF-A*	TGTGTGTGTGTGAGTGGTTGA	TCTCTGTGCCTCGGGAAG
*WNT1*	CGCTGGAACTGTCCCACT	AACGCCGTTTCTCGACAG

## Data Availability

The data presented in this study are available on request from the corresponding author.
